# Second primary colorectal cancer after the initial primary colorectal cancer

**DOI:** 10.1186/s12885-018-4823-6

**Published:** 2018-09-27

**Authors:** Lin Yang, Zhenchong Xiong, Qian Kun Xie, Wenzhuo He, Shousheng Liu, Pengfei Kong, Chang Jiang, Xiaojun Xia, Liangping Xia

**Affiliations:** 1 Sun Yat-sen University Cancer Center, 651 Dongfeng Road east, Guangzhou, 510060 China; 2 State Key Laboratory of Oncology in Southern China, Guangzhou, China; 3 Collaborative Innovation center for Cancer Medicine, Guangzhou, China

**Keywords:** Second primary colorectal cancer (SPCRC), Standardized incidence ratio (SIR), Left-sided colon cancer (LCC), Right-sided colon cancer (RCC), Overall survival (OS)

## Abstract

**Background:**

Initial primary colorectal cancer (IPCRC) has a high risk of developing into second primary colorectal cancer (SPCRC). Right-sided colon cancer (RCC) and left-sided colon cancer (LCC) have different characteristics and are considered to be two different entities. However, the different risks for SPCRC in categorized tumor sites and SPCRC subcategorized sites have not been fully elucidated to date. We aimed to compare incidence and survival of IPCRC and SPCRC and characterize the risk factors of SPCRC while also comparing the different SPCRC characteristics.

**Methods:**

We used the National Cancer Institute’s Surveillance, Epidemiology, and End Results (SEER) data to compute standardized incidence ratios (SIR) in order to estimate risk of SPCRC after IPCRC diagnosis. The most prominent risk factors for SPCRC were measured by multivariate regression analysis and the temporal trend of SPCRC incidence was assessed with Joinpoint regression. Survival of patients with SPCRC and IPCRC was compared by Kaplan-Meier analysis.

**Results:**

Patients with IPCRC were 1.73 times more likely to develop SPCRC (SIR = 1.73, 95% CI 1.69–1.78). SPCRC incidence declined since the first 8 years of IPCRC diagnosis to baseline. We demonstrated poorer survival with SPCRC compared with IPCRC while second RCC resulted in better survival compared with second LCC. Black ethnicity, age range 70–79, and LCC were associated with the highest risk of developing SPCRC.

**Conclusion:**

The characteristic differences between second LCC and RCC were relatively narrow. Furthermore, in those with SPCRC, RCC had the best survival outcome.

**Electronic supplementary material:**

The online version of this article (10.1186/s12885-018-4823-6) contains supplementary material, which is available to authorized users.

## Background

Colorectal cancer (CRC) is the third most documented malignancy in men and women and the second most common cause of cancer morbidity [1]. In the United States of America, 13,543 new cases of CRC were diagnosed in 2017 [2] while the estimated number of survivors with colorectal cancer was approximately 85,170 as of 2017 [2]. The number of survivors of colorectal cancer is expected to rapidly increase partly because of changes to living habits (e.g. decreased smoking incidence and red meat consumption), the adoption of general screening tests, early detection tests, and improvements in treatment [3].

Recent studies using the National Cancer Institute’s Surveillance, Epidemiology, and End Results (SEER) database has shown a statistically significant increase (approximately 1.4-fold) in the risk of subsequent primary cancers in patients with CRC compared with the general population [4–7]. The risk increase in these study was not elevated sufficiently to alter colorectal screening practice and the risk trend was also not illuminated. Additionally, second primary CRC sites characteristics and outcomes difference of had not reported.

Right-sided colon cancer (RCC) and lefts-sided colon cancer (LCC) differences have been noted [8–10]. However, it is unclear whether these biologic differences translate into meaningful clinical differences in second cancer. Rectal cancer is the second leading cause of cancer-related deaths in the USA and constitutes approximately 28% of all large bowel carcinomas. Although rectal cancer is more commonly seen in the elderly, recent studies have shown an increased incidence of rectal cancer in adults < 40 years old [11]. Due to longer survival rates and increasing proportions of younger incident patients [12], second cancers are important to consider [11, 13].

Second primary malignancies (SPMs) account for between 17 to 19% of incident cancer cases [14, 15]. The risk of SPM in patients with cancer is multifactorial but is related to genetic predisposition, treatment-related malignant transformations, and/or continuing exposure to the etiologic agents [16]. It has been estimated that by 2026, there will be > 20 million cancer survivors in the United States at risk of SPMs [17].

The purpose of this study was to describe the effect of the initial primary colorectal cancer (IPCRC) on the incidence, stage at diagnosis, and tumor site characteristics that were different for subsequent second primary colon-rectal cancers (SPCRC). In addition, we sought to describe the stage at presentation and outcomes of SPCRCs occurring in patients with a prior diagnosis of IPCRC.

## Methods

### Data source

This study used a general dataset that selected data from a public health program and therefore did not require institutional review board approval. We identified patients with diagnosed CRC reported by the Surveillance, Epidemiology, and End Results (SEER) program of the National Cancer Institute between 1992 and 2012.

All cases of IPCRC were: histologically confirmed colorectal cancer and pathological type is adenocarcinoma or mucinous adenocarcinoma [18]. Cases where diagnosis of IPCRC was confirmed by death certificate only or autopsy only were excluded. A minimum interval of 6 months between the diagnoses was required to exclude synchronous cancers (latency period).

Three sites were evaluated: the right colon (cecum, ascending colon, hepatic flexure, and transverse colon); the left colon (splenic flexure, descending colon, and sigmoid colon), and the rectosigmoid colon (rectosigmoid junction and rectum). The SEER stage included localized (malignant but confined to primary organ), regional (invasion beyond primary organ but no distant metastasis), or distant (with distant metastasis) and unknown [19]. The study cohort was divided into subgroups based on latency intervals: 0–4 years, 4–8 years, > 8 years. Individuals within each cohort (e.g. LCC, RCC, rectosigmoid cancer) were classified by whether they had an ensuing diagnosis of second primary malignant neoplasm of CRC, hereafter referred to as SPCRC.

### Observed survival

Survival time was defined as the number of months from study commencement until the date of death from any cause, or until the end of the observation period. Patients alive at the end of follow-up were censored.

### Statistical methods

Standardized incidence ratios (SIRs) and absolute excess risk (AER) values were obtained from the SEER program using SEER*Stat software. SIRs were computed by comparing the observed (O) number of second malignancies with those expected (E) in the general population to quantify second malignancy risks to assess excess relative risk of SPCRC compared with the general population. Within SEER, the AER is defined as the excess cancers per 10,000 persons per year, which is calculated as follows: ([observed count-expected count] × 10,000)/ person-years at risk [20]. Joinpoint regression was used to analyze SPCRC incidence and mortality risk trends. We use annual percent change (APC) to assess rate changes.

Multivariable logistic regression was used to determine which prognostic factor were associated with the presence of SPCRC at diagnosis. The Kaplan-Meier method was used to compare survival outcomes.

A *P* value of 0.05 was considered statistically significant and *P* values were two sided. Analyses were performed with the SAS version 9.2 software package (SAS Institute, Cary, North Carolina) and the survival package within the R version 2.11 statistical software package (http://www.r-project.org) was used.

## Results

### Study population

After exclusions, together, among all the IPCRCs, 2.0% (4977/252,404) were considered to have 2nd CRC, and the incidence in those with RCC was 2.3% (1756/96,754), 2.3% (1770/73,132) in those with LCC, and 1.8% in those with rectal cancer (1451/77,550) (Additional file 1: Table S1). Those aged between 70 and 79 years had the highest proportion (31.8%) of SPCRC and this was the same for other CRCs (35.4% for RCC, 31.2% for LCC, and 28.3% for rectosigmoid cancer). Based on the SEER staging system, 1989 (40.0%) SPCRCs were considered to be localized disease, 1703 (34.3%) had regional disease, 888 (17.8%) had metastatic lesions, and the remainder (8.0%) of the patients unstated. When we analyzed the 2nd CRC distribution by histology grade, we found that moderately differentiated cancer accounted for the largest proportion of SPCRC, which was apparently higher than any of the other histology groups. More than half of 2nd CRCs were diagnosed in the first four years in but approximately 20% of 2nd CRCs were identified in the following 4–8 years after the 1st CRC. Patients with surgery history (95.1%) were more likely to develop SPCRC compared to patients without surgery history (4.9%). For patients without surgery, they are more likely to be in the early stage and had better prognosis, then the chance to have the SPCRC is bigger. However, for patients without surgery history, they may tend to be in the advanced stage and the prognosis is poor, so the probability to have the SPCRC is relatively reduced.

Table 1 compares the demographic and clinical features of second primary CRC between RCC, LCC, and rectal cancer. RCC accounted for nearly half of these SPCRCs (2144/4977). Patients tend to have more localized disease, be of white ethnicity and have moderately differentiated disease at diagnosis of the second primary RCC. The median time to the 2nd RCC, LCC, and rectosigmoid cancer was 38.0 months, 28.0 months, and 31.0 months, respectively. The mean age exceeded 70 years of age and the eldest was in the RCC cohort.

**Table 1:** The characteristic differences in the SPCRC

Site group	RCC	LCC	*P* value	Rectal cancer
Number	2144 (43.1%)	1262 (25.4%)		1430 (28.7%)
Mean Age development of 2nd cancer (years)	76.0	74.0	0.146	70.0
Sex			0.113	
Male	1017 (48.1%)	689 (54.6%)		813 (56.9%)
Female	1097 (51.9%)	573 (45.4%)		617 (43.1%)
Race				
Black	236 (11.2%)	147 (11.6%)		190 (13.3%)
White	1563 (73.9%)	873 (69.2%)		882 (61.7%)
Hispanic/Latino	141 (6.7%)	115 (9.1%)		172 (12.0%)
Asian or Pacific Islander and others	173 (8.2%)	126 (10.0%)		182 (12.7%)
Unknown	1 (0.1%)	1 (0.1%)		4 (0.3%)
SEER staging				
Localized	1066 (50.%)	558 (44.2%)	0.061	660 (46.2%)
Regional	670 (31.7%)	373 (29.6%)		341 (23.8%)
Distant	245 (11.6%)	218 (17.3%)		218 (15.2%)
Unknown	133 (6.3%)	113 (9.0%)		211 (14.8%)
Surgery				
No	219 (10.4%)	180 (14.3%)		405 (28.3%)
Yes	1892 (89.5%)	1079 (85.5%)		1018 (71.2%)
Unknown				7 (0.5%)
Pathology grade			0.312	
Well differentiated	200 (9.5%)	127 (10.1%)		115 (8.0%)
Moderately differentiated	1200 (56.8%)	749 (59.4%)		769 (53.8%)
Poorly differentiated	422 (20.0%)	169 (13.4%)		172 (12.0%)
Undifferentiated	27 (1.3%)	13 (1.0%)		11 (0.8%)
Unknown	265 (12.5%)	204 (16.2%)		363 (25.4%)
Calendar year				
1992–2002	801 (37.9%)	490 (38.8%)		460 (32.2%)
2003–2012	1313 (62.1%)	772 (61.2%)		970 (67.8%)
Median time to secondary cancer (months)	38.0	28.0		31.0

Abbreviations: SPCRC, secondary primary colorectal cancer; RCC, right-sided colon cancer; LCC, left-sided colon cancer. The P values are the difference of the SPCRC between RCC and the LCC

### SPCRC trends

**Fig. 1:**
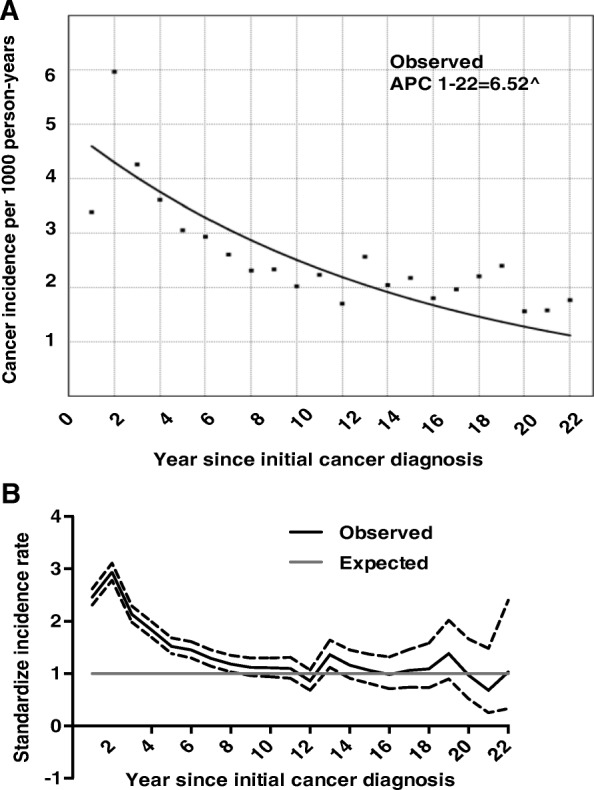
Temporal trend of second primary colorectal cancer (SPCRC) incidence over a 22-year follow-up period in the United States from 1992 to 2012. (**a**). Trend of SPCRC incidence per 1000 person-years is estimated by Joinpoint regression (Trend 1: 1st-8th year, Trend 2: 8th–20th year). ^ *P* < 0.05. (**b**) Trend of SIR (standardized incidence ratio) SPCRC in the colorectal cancer (CRC) compared with the general population. The X-axis represents the intervals of follow-up and the Y-axis represents rates per 100,000 of the US population. The dotted line means that the 95% CI

Figure 1 shows the trends in incidence rate of CRC in the standard US population and the trend in the excess risk of a second primary CRC following latency time. SPCRC incidence has declined since the first year of the diagnosis of the IPCRC (Fig. 1a). Compared with the general population, the risk summit for SPCRC appears in the second year of the IPCRC diagnosis in the study cohort (Fig. 1b). In the first 8-years during follow-up, the risk of second CRC cancer patients was significantly higher compared with the general population and then tended to have similar likelihood as that of the general population with intervals of > 8 years for diagnosis (Fig. 1b). Similar results were obtained focusing on LCC, RCC, and primary rectal cancers (Additional file 1: Figure S1).

### Identifying factors associated with SPCRC risk

Overall CRC risk was significantly higher than expected in the general population after CRC (SIR, 1.73; 95% CI, 1.69–1.78), RCC (SIR, 1.52; 95% CI 1.45–1.59), LCC (SIR, 1.98; 95% CI, 1.89–2.08), and rectosigmoid cancer (SIR, 1.77; 95% CI, 1.67–1.86) (Additional file 1: Table S2). The calculated excess risk (beyond the expected number) was 15.38 per 100,000 in the whole cohort, 12.09 in the RCC, 20.33 for the LCC, and 14.21 for rectosigmoid cancer. The resulting SIR data and AER values are given in Additional file 1: Table S2. Furthermore, the 2nd CRC malignancies risk was elevated for LCC (SIR, 1.73; 95% CI, 1.64–1.83), RCC (SIR, 1.65; 95% CI, 1.56–1.72), and rectosigmoid cancer (SIR, 2.04; 95% CI, 1.94–2.15). Stratified analyses suggested that the risk might be a consistent phenomenon across almost all other subtypes. Of note, patients diagnosed at younger ages (< 40 years) were particularly more prone to 2nd CRC (SIR, 14.78; 95% CI, 12.20–17.74 for the 2nd CRC; SIR, 15.15; 95% CI, 10.49–21.17 for the 2nd RCC; SIR, 10.42; 95% CI, 6.67–15.50 for the 2nd LCC; SIR, 17.88; 95% CI, 13.54–23.16 for the 2nd rectosigmoid cancer).

**Fig. 2:**
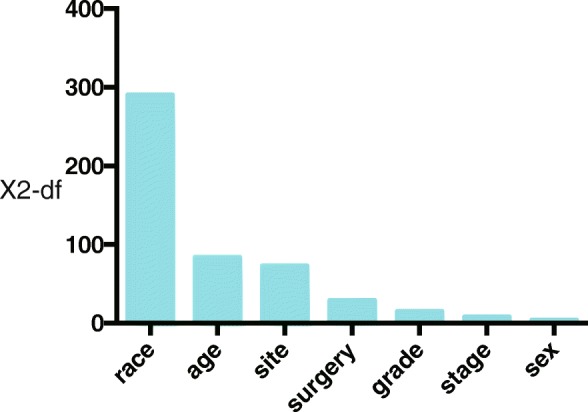
Importance of variables in predicting the risk of second primary colorectal cancer (SPCRC). The y-axis shows the likelihood ratio test x^2^ statistic subtracted by the degrees of freedom (df) conducted for each variable. The importance metric was displayed for the main effects of the variables in the multivariable model

As is shown by the multivariate Cox regression in the Table 2, History of LCC (LCC vs RCC, HR = 1.369, 95% CI, 1.269–1.477, *P* < 0.001), History of rectosigmoid colon cancer (rectosigmoid colon cancer vs RCC, HR = 1.090, 95% CI, 1.003–1.184), age > 60 (60–69 vs < 40, HR = 1.342, 95% CI, 1.073–1.679, *P* = 0.010; 70–79, HR = 1.541, 95% CI, 1.234–1.925, *P* < 0.001; > 80 vs < 40, HR = 1.339, 95% CI, 1.066–1.680, *P* = 0.012), Female (male vs female, HR = 0,930, 95% CI, 0.871–0.992, *P*-0.028), Black (White vs Black, HR = 0.490. 95% CI, 0.447–0.536, *P* < 0.001; Hispanic/Latino, HR = 0.604, 95% CI, 0.528–0.690, *P* < 0.001; Asian and Pacific, HR = 0.798, 95% CI, 0.710–0.898, *P* < 0.001), without surgery history (No vs yes, HR = 1.657, 95% CI, 1.383–1.985, *P* < 0.001), well differentiated pathology (moderately vs well, HR = 0.868, 95% CI, 0.785–0.961, *P* = 0.006; poorly vs well, HR = 0.814, 95% CI, 0.720–0.919, *P* = 0.001; undifferentiated vs ell, HR = 0.524, 95% CI, 0.354–0.775, *P* = 0.001) were the risk factors of the presence of SPCRC. Figure 2 shows the relative importance of each independent variable and shows that race in ISPCRC diagnosis was the most important factor in predicting SPCRC risk, followed by age at diagnosis and primary tumor site. Compared with a reference race of black ethnicity, survivors of other race had substantially reduced risks of SPCRC, with a hazard ratio (HRs) < 0.799. Compared with those aged < 40 years, the age group 70–79 had the highest risk of developing SPCRC (HR = 1.516, *P* < 0.001). Those with LCC had an increased risk of SPCRC compared with a reference RCC (HR = 1.362, *P* < 0.001) and the trend still remained after accounting for other confounding factors.

**Table 2:** Factors Associated With Secondary Primary CRC (SPCRC) Risk Among Survivors of colorectal cancer

Cancer type	Univariate Cox Regression			Multivariate Cox Regression		
	HR	95% CI	*P*	HR	95% CI	*P*
History of IPCRC			< 0.001			< 0.001
RCC	1.000	1.000		1.000	1.000	
LCC	1.362	1.264–1.468	< 0.001	1.369	1.269–1.477	< 0.001
Rectosigmoid colon	1.031	0.951–1.118	< 0.454	1.090	1.003–1.184	0.041
Age at diagnosis			< 0.001			< 0.001
< 40	1.000	1.000		1.000	1.000	
40–49	1.026	0.800–1.315	0.839	1.018	0.794–1.305	0.890
50–59	1.027	0.816–1.293	0.820	0.954	0.799–1.268	0.954
60–69	1.352	1.082–1.691	0.008	1.342	1.073–1.679	0.010
70–79	1.516	1.215–1.892	< 0.001	1.541	1.234–1.925	< 0.001
> 80	1.257	1.003–1.576	0.047	1.339	1.066–1.680	0.012
Sex						
Female vs. Male	0.928	0.870–0.989	0.021	0.930	0.871–0.992	0.028
Race			< 0.001			< 0.001
Black	1.000	1.000		1.000	1.000	< 0.001
White	0.490	0.447–0.536	< 0.001	0.490	0.447–0.536	< 0.001
Hispanic/Latino	0.581	0.508–0.664	< 0.001	0.604	0.528–0.690	< 0.001
Asian or Pacific Islander and others	0.799	0.710–0.899	< 0.001	0.798	0.710–0.898	< 0.001
SEER staging			0.001			0.007
Localized	1.000	1.000		1.000	1.000	
Regional	0.947	0.882–1.017	0.159	0.943	0.878–1.013	0.109
Distant	1.116	1.023–1.116	0.013	1.087	0.996–1.186	0.061
If surgery						
Yes vs. No	1.685	1.409–2.016	< 0.001	1.657	1.383–1.985	< 0.001
Pathology grade			< 0.001			< 0.001
Well differentiated	1.000	1.000		1.000	1.000	
Moderately differentiated	0.871	0.787–0.963	0.007	0.868	0.785–0.961	0.006
Poorly differentiated	0.775	0.686–0.875	< 0.001	0.814	0.720–0.919	0.001
Undifferentiated	0.487	0.329–0.719	< 0.001	0.524	0.354–0.775	0.001

Abbreviations: SPCRC, secondary primary colorectal cancer; RCC, right-sided colon cancer; LCC, left-sided colon cancer

### Observed survival (OS) comparison

**Fig. 3:**
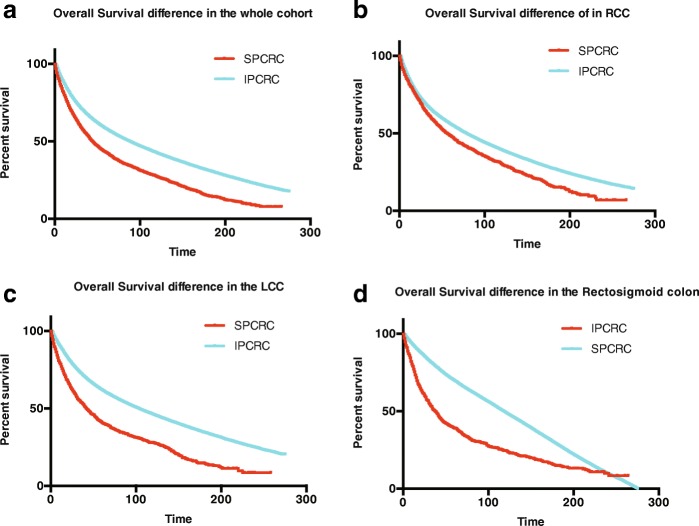
Initial primary colorectal cancer (IPCRC) and second primary colorectal cancer (SPCRC) overall survival (OS) difference in individuals from the (**a**) whole cohort, (**b**) those with right-sided colon cancer (RCC), (**c**) those with left-sided colon cancer (LCC), and those with (**d**) rectal cancer

**Fig. 4:**
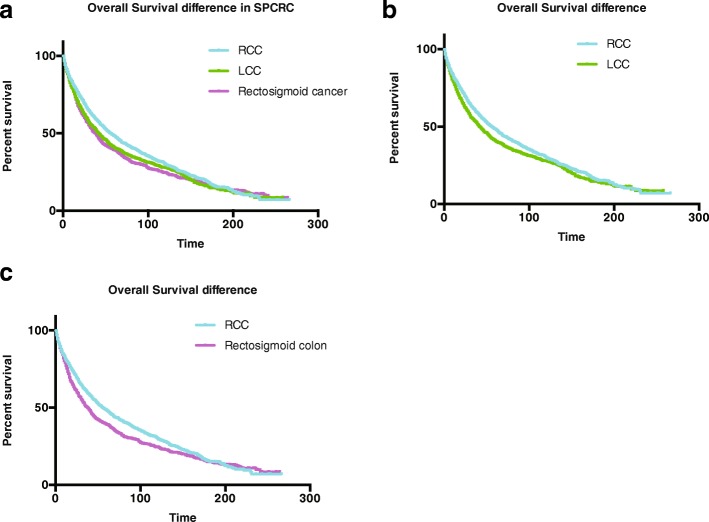
Second primary colorectal cancer (SPCRC) overall survival (OS) difference in individuals from the (**a**) whole cohort, (**b**) those with right-sided colon cancer (RCC) vs. those with left-sided colon cancer (LCC), (**c**) right-sided colon cancer (RCC) vs. Rectosigmoid cancer, and (**d**) left-sided colon cancer (LCC) vs. rectosigmoid cancer

We used follow-up data to compute actual observed survival estimates comparing patients with SPCRC and those with IPCRC. The estimates are shown in Fig. 3. The survival estimates for the IPCRC and the SPCRC groups were different (log-rank, P < 0.001). We generated separate OS comparison data for categorized sites. For RCC, LCC, and rectosigmoid cancer, those with SPCRC had an apparent poorer survival outcome than the IPCRC groups (*P* < 0.001 for RCC, LCC, and rectosigmoid cancer, respectively, Fig. 3). We then further compared the OS between those with SPCRC by cancer sites and we found that those with RCC had the best survival outcome (*P* < 0.001for the whole cohort; *P* = 0.002 for RCC vs LCC; *P* < 0.001 for RCC vs. rectosigmoid cancer; and *P* = 0.352 for LCC and rectosigmoid cancer) (Fig. 4).

## Discussion

These data provide an update of earlier studies with additional data on tumor site and survival. Compared with a reference of 2nd RCC, survivors of LCC had substantially increased risks of SPCRC. Furthermore, although the characteristic differences between the 2nd RCC and the 2nd LCC was narrowed, those with 2nd RCC had the best survival outcome.

The association between initial colon cancer and 2nd colon cancer has been reported by Ananya et al. where the relative incidence rate of second primary CRC increased during the 1990s [7] but that study did not examine the survival outcome differences or the SPCRC categorical site differences. The findings of the current study indeed confirm that IPCRC had elevated risk (SIR = 1.73 and AER = 15.38) for SPCRC and this was the same for 2nd RCC, LCC, and rectosigmoid cancer. In the study by Green et al., the incidence of second primary CRC remained high despite intensive surveillance strategies [21]. This may be attributed that the patients with a primary CRC may be biologically different from the general population and at a much higher risk of developing subsequent cancers. A correlation between the hypothetical molecular basis of SPCRC and tumor location in the colon in the same individual has been proposed [22]. SPCRC arising exclusively in the right colon appears to be related mainly to the Lynch syndrome (LS) or CIMP pathways. When SRCRC is located in the left colon, the CIN pathway may be associated with a contribution of lower penetrance genes. Finally, cases with SPCRC throughout the entire colon appear to be related to the CIN pathway due to germline mutations in APC or MUTYH genes.

Although the LCCs account for the biggest proportion in the IPCRC, we found that within this SPCRC location group, tumors were mainly the right colon tumors. Furthermore, the clinical differences in the initial primary LCC and the RCC were narrowed. In this study, we found that that there was no difference with respect to gender, age, pathology grade between the 2nd RCC and 2nd LCC, while the SPCRC tended to be diagnosed in the early stage of disease [23, 24]. Several studies have explored potential differences in overall survival between initial primary RCC and LCC, although there is some contention to the findings of such studies. However, there have been no studies that have investigated the 2nd LCC and RCC survival difference. In this study, we found that 2nd RCC had an apparently improved survival rate than those with 2nd LCC. This may due to the survival differences at the tumor site and also in the tumor features. In this study, there was a narrowing in the poorer prognostic characteristics of RCC, such as older age, more advanced tumor stage, and undifferentiated pathology. Such parameters have a much greater effect on survival in patients with CRC. The RCC tend to be more often in advanced tumor stage compared with the LCC and patients with LCC had less frequently locally advanced tumors (T4 stage) [25–27]. This might explain the reason that 2nd cancer was more often identified in RCC but not in LCC.

Incidence of SPCRC varies from person to person and identifying a high-risk population of developing SPCRC is important for optimal surveillance and management in colorectal cancer patients. Our study showed that the incidence of SPCRC was continuously increasing within 2 years after the first diagnosis of CRC, and it gradually approached baseline in the 8 years after the first diagnosis. These results suggested that CRC patients have an excess risk of SPCRC over a period of 8 years, which is of great importance. We discovered that patients with CRC had double the risk of SPCRC compared with the general population to develop the CRC and the risk hazards remained in the subgroups.

Using multivariate regression, black ethnicity, aged > 60, LCC cohort, treatment with surgery, well-differentiated disease, SEER distant staging, and male gender were all significant risk factors for developing SPCRC. Colon cancer may occur at a significantly younger age in black patients compared to white patients, with black patients having approximately double the proportion of colon cancers diagnosed before the age of 50 (10.6% vs 5.5%) [28]. The socioeconomic and sociodemographic status disparities might account for the high risk of 2nd CRC in those of black ethnicity. The association between young age and lower risk of several second cancers has been reported previously and our data found the highest risk of developing the second CRCs to be in those aged 70–79, which is consistent with previous studies [7, 29, 30]. Although this finding appears rather counter-intuitive since young survivors have more time to develop SPCRC and thus could have a higher risk of SPCRC, it is possible that this association exists partly because the older patients have a higher probability of developing invasive cancer, where this occurs for 1 in 3 men and for 1 in 4 women [31]. Thus, the probability of co-occurrence of SPCRC in the same patient rises with increasing age. The LCC cohort and patients who had surgery have a high risk of developing 2nd CRC, and it may be that the LCC cohort and those treated with surgery have better survival outcomes than the RCC cohort, while also having more chances of developing SPCRC.

There are several limitations to this study. We cannot exclude the possibility that some of these patients with a second primary CRC may represent hereditary nonpolyposis colon cancer (HNPCC) but patients with familial polyposis syndromes are mostly diagnosed with CRC before the age of 50 yr. [32]. However, in our analysis, we found that the proportion of SPCRC in patients aged younger than 50 years was less than 10%. Furthermore, HNPCC accounts for only 1–5% of patients diagnosed with colon cancer and is not likely to significantly bias our observations.

In summary, this is the first study to analyze a large population-based cancer registry to quantify SPCRC survival difference with the IPCRC and the risk factors of SPCRC. The relative risk of SPCRC appears to have increased with black ethnicity, age > 60 and LCC in the first 8 years of IPCRC diagnosis. Moreover, those with 2nd RCC had the best survival outcome.

## Conclusion

These data provide an update of earlier studies with additional data on tumor site and survival of SPCRC. Compared with a reference race of 2nd RCC, survivors of LCC had substantially increased risks of SPCRC. Furthermore, in those with SPCRC, the RCC cohort was more likely to survive.

## Additional file

Additional file 1: **Table S1.** Clinical Characteristics of Patients with and without a SPCRC. **Table S2.** Standardized Incidence Ratios and Absolute Excess Risk of a SPCRC. **Figure S1.** Temporal trend of second primary colorectal cancer (SPCRC) incidence over the 22-year follow-up period (A). Trend of SPCRC incidence per 1000 person-years is estimated by Joinpoint regression. ^ *P* < 0.05. The solid black line shows the trend in (1) right-sided colon cancer, RCC; (2) left-sided colon-cancer, LCC (3) Rectosigmoid cancer in the United States from 1992 to 2012. The X-axis represents the intervals of follow-up and the Y-axis represents rates per 100,000 of the US population. (DOCX 214 kb)

## Abbreviations

AER: absolute excess risk
CIMP: CpG island methylator phenotype
CIN: Chromosomal instability
CRC: Colorectal cancer
HNPCC: hereditary nonpolyposis colon cancer
IPCRC: Initial primary colorectal cancer
LS: Lynch syndrome pathway
MSI: microsatellite instability
MSI: Microsatellite instability
MSS: Microsatellite stability
OS: Overall survival
SEER: Surveillance, Epidemiology, and End Results
SIR: Standardized incidence ratio
SPM: Second primary malignancies
