# Supplementary material for: Second primary colorectal cancer after the initial primary colorectal cancer
Source: BMC Cancer. 2018 Sep 27;18:931. doi: 10.1186/s12885-018-4823-6 (PMC6161401; doi:10.1186/s12885-018-4823-6)
Supplement: Supplementary file 1 — Table S1. Clinical Characteristics of Patients with and without a SPCRC. Table S2. Standardized Incidence Ratios and Absolute Excess Risk of a SPCRC. Figure S1. Temporal trend of second primary colorectal cancer (SPCRC) incidence over the 22-year follow-up period (A). Trend of SPCRC incidence per 1000 person-years is estimated by Joinpoint regression. ^ P < 0.05. The solid black line shows the trend in (1) right-sided colon cancer, RCC; (2) left-sided colon-cancer, LCC (3) Rectosigmoid cancer in the United States from 1992 to 2012. The X-axis represents the intervals of follow-up and the Y-axis represents rates per 100,000 of the US population. (DOCX 214 kb) [file 12885_2018_4823_MOESM1_ESM.docx]

**Table S1. Clinical Characteristics of Patients with and without a SPCRC**

| **Patient Characteristics** | **Full cohort**  **(N=259,072)** | | **RCC**  **(N=98,501)** | | **LCC**  **(N=74,902)** | | **Rectosigmoid colon (N=79,001)** | |
| --- | --- | --- | --- | --- | --- | --- | --- | --- |
|  | **Without SPCRC** | **With SPCRC** | **Without SPCRC** | **With SPCRC** | **Without SPCRC** | **With SPCRC** | **Without SPCRC** | **With SPCRC** |
| **Age at diagnosis (years)** | 247,427 | 4977 | 96754 | 1,756 | 73,132 | 1770 | 77,550 | 1451 |
| <40 | 7,525 (3.0%) | 118  (2.4%) | 2,387 (2.5%) | 33 (1.9%) | 2,052 (2.8%) | 43 (2.4%) | 3,086 (4.0%) | 42 (2.9%) |
| 40-49 | 20,417 (8.3%) | 337 (6.8%) | 5,656 (5.8%) | 106 (6.0%) | 6,316 (8.6%) | 118 (6.7%) | 8,445 (10.9%) | 113 (7.8%) |
| 50-59 | 46,348 (18.7%) | 738 (14.8%) | 13,353 (13.8%) | 205 (11.7%) | 14,748 (20.2%) | 255 (14.4%) | 18,247 (23.5%) | 278 (19.2%) |
| 60-69 | 59,833 (24.2%) | 1,281 (25.7%) | 21,424 (22.1%) | 409 (23.3%) | 18,917 (25.2%) | 488 (27.6%) | 19,492 (25.1%) | 384 (26.5%) |
| 70-79 | 65,436 (26.4%) | 1,584 (31.8%) | 28,749 (29.7%) | 622 (35.4%) | 19,156 (26.2%) | 552 (31.2%) | 17,531 (22.6%) | 410 (28.3%) |
| >80 | 47,868 (19.3%) | 919 (18.5%) | 25,176 (26.0%) | 381 (21.7%) | 11,943 (16.3%) | 314 (17.7%) | 10,749 (13.9%) | 224 (15.4%) |
| **Sex** |  |  |  |  |  |  |  |  |
| Male | 125,800 (50.8%) | 2596 (52.2%) | 43,445 (44.9%) | 809 (46.1%) | 38,525 (52.7%) | 977 (55.2%) | 43,830 (56.5%) | 810 (55.8%) |
| Female | 121,627 (49.2%) | 2381 (47.8%) | 53,300 (55.1%) | 947 (53.9%) | 34,607 (47.3%) | 793 (44.8%) | 33,720 (43.5%) | 641 (44.2%) |
| **Race** |  |  |  |  |  |  |  |  |
| Black | 23,144 (9.4%) | 806 (16.2%) | 9,130 (9.4%) | 288 (16.4%) | 6,885 (9.4%) | 283 (16.0%) | 7,129 (9.2%) | 235 (16.2%) |
| White | 176,096 (71.2%) | 2981  (59.9%) | 70,228 (72.6%) | 1,026 (58.4%) | 51,545 (70.5%) | 1,104 (62.4%) | 54,323 (70.0%) | 851 (58.6%) |
| Hispanic/Latino | 22,318 (9.0%) | 441 (8.9%) | 7,790 (8.1%) | 138 (7.9%) | 6,670 (9.1%) | 152 (8.6%) | 7,848 (10.1%) | 151 (10.4%) |
| Asian or Pacific Islander and others | 24,580 (9.9%) | 688  (13.8%) | 9,110 (9.4%) | 279 (15.9%) | 7,622 (10.4%) | 214 (12.1%) | 392 (0.5%) | 195 (13.4%) |
| Unknown | 1,289 (0.5%) | 61 (1.2%) | 487 (0.5%) | 25 (1.4%) | 410 (0.6%) | 17 (1.0%) |  | 19 (1.3%) |
| **SEER staging** |  |  |  |  |  |  |  |  |
| Localized | 103,050 (41.6%) | 1989 (40.0%) | 40,539 (41.9%) | 685 (39.0%) | 30,281 (41.4%) | 703 (39.7%) | 32230 (41.6%) | 601 (41.4%) |
| Regional | 93,062 (37.6%) | 1,703 (34.2%) | 36,194 (37.4%) | 580 (33.0%) | 27,569 (37.7%) | 622 (35.1%) | 29,299 (37.8%) | 501 (34.5%) |
| Distant | 41,921 (16.9%) | 888 (17.8%) | 16,374 (16.9%) | 331 (18.8%) | 12,458 (17.0%) | 317 (17.9%) | 13,089 (16.9%) | 240 (16.5%) |
| Unknown | 9,394 (3.8%) | 397 (8.0%) | 3,638 (3.8%) | 160 (9.1%) | 2,824 (3.9%) | 128 (7.2%) | 2,932 (3.8%) | 109 (7.5%) |
| **Surgery** |  |  |  |  |  |  |  |  |
| No | 19,883 (8.0%) | 242 (4.9%) | 5,283 (5.5%) | 47 (2.7%) | 3,980 (5.4%) | 67 (3.8%) | 10,620 (13.7%) | 128 (8.8%) |
| Yes | 227,544  (92.0%) | 4,735 (95.1%) | 91,441 (94.5%) | 1709 (97.3%) | 69,132 (94.5%) | 1703 (96.2%) | 66,851 (86.2%) | 1,323 (91.2%) |
| Unknown |  |  | 21 (<0.1% |  | 20 (<0.1%) |  | 79 (0.1%) |  |
| **Marital status** |  |  |  |  |  |  |  |  |
| Single, Separated, Divorced, | 52,445 (21.2%) | 1,012  (20.3%) | 19,428 (20.1%) | 350 (19.9%) | 15,346 (21.0%) | 355 (20.1%) | 17,671 (22.8%) | 307 (21.2%) |
| Married (including common law) | 138,625 (56.0%) | 2,776 (55.8%) | 51,755 (53.5%) | 941 (53.6%) | 42,223 (57.7%) | 991 (56.0%) | 44,647 (57.6%) | 844 (58.2%) |
| Widowed | 45,921 (18.6%) | 997 (20.0%) | 22,271 (23.0%) | 411 (23.4%) | 12,241 (16.7%) | 342 (19.3%) | 11,409 (14.7%) | 244 (16.8%) |
| Unknown | 10,436 (4.2%) | 192 (3.9%) | 3,291 (3.4%) | 54 (3.1%) | 3,322 (4.5%) | 82 (4.6%) | 3,823 (4.9%) | 56 (3.9%) |
| **Pathology grade** |  |  |  |  |  |  |  |  |
| Well differentiated | 22,117 (8.9%) | 514  (10.3%) | 8,249 (8.5%) | 165 (9.4%) | 7,386 (10.1%) | 217 (12.3%) | 6,482 (8.4%) | 132 (9.1%) |
| Moderately differentiated | 150,481 (60.8%) | 3060  (61.5%) | 57,086 (59.0%) | 1,050 (59.8%) | 47,702 (65.2%) | 1,145 (64.7%) | 45,693 (58.9%) | 865 (59.6%) |
| Poorly differentiated | 40,364 (16.3%) | 752 (15.1%) | 20,974 (21.7%) | 374 (21.3%) | 9,015 (12.3%) | 219 (12.4%) | 10,375 (13.4%) | 159 (11.0%) |
| Undifferentiated | 2,692 (1.1%) | 28 (0.6%) | 1,549 (1.6%) | 18 (1.0%) | 541 (0.7%) | 5 (0.3%) | 602 (0.8%) | 5 (0.3%) |
| Unknown | 31,773 (12.8%) | 623 (12.5%) | 8,887 (9.2%) | 149 (8.5%) | 8,488 (11.6%) | 184 (10.4%) | 14,398 (18.6%) | 290 (20.0%) |
| **Calendar year** |  |  |  |  |  |  |  |  |
| 1992-2002 | 130,889 (52.9%) | 3167 (63.6%) | 50,150 (51.8%) | 1,129 (64.3%) | 40,376 (55.2%) | 1166 (65.9%) | 40,363 (52.0%) | 872 (60.1%) |
| 2003-2012 | 116,538 (47.1%) | 1810 (36.4%) | 46,595 (48.2%) | 627 (35.7%) | 32,756 (44.8%) | 640 (34.1%) | 37,187 (48.0%) | 579 (39.9%) |
| **Latency time** |  |  |  |  |  |  |  |  |
| 0-4 |  | 3,176 (63.8%) |  | 1135 (64.6%) |  | 1149 (64.9%) |  | 892 (61.5%) |
| 4-8 |  | 1,068 (21.5%) |  | 364 (20.7%) |  | 363 (20.5%) |  | 341 (23.5%) |
| >8 |  | 733 (14.7%) |  | 257 (14.6%) |  | 258 (14.6%) |  | 218 (15.0%) |

Abbreviation: SPCRC, secondary primary colorectal cancer; RCC, right-sided colon cancer; LCC, left-sided colon cancer.

**Table S2. Standardized Incidence Ratios and Absolute Excess Risk of a SPCRC**

| **Patient Characteristics** | **Full cohort**  **(N=259,072)** | | **RCC (N=98,859)** | | **LCC (N=74,394)** | | **Rectosigmoid colon (N=78,980)** | |
| --- | --- | --- | --- | --- | --- | --- | --- | --- |
|  | **AER** | **SIR (95%CI)** | **AER** | **SIR (95%CI)** | **AER** | **SIR (95%CI)** | **AER** | **SIR (95%CI)** |
| **Whole study** | 15.38 | 1.73 (1.69-1.78) | 6.07 | 1.65 (1.56-1.72) | 3.93 | 1.73 (1.64-1.83) | 5.31 | 2.04 (1.94-2.15) |
| **Age group** |  |  |  |  |  |  |  |  |
| <40 | 22.51 | 14.78 (12.20-17.74) | 6.49 | 15.15 (10.49-21.17) | 4.44 | 10.42 (6.67-15.50) | 11.0 | 17.88 (13.54-23.16) |
| 40-49 | 20.96 | 5.27 (4.71-5.87) | 7.59 | 6.35 (5.27-7.59) | 4.89 | 4.37 (3.48-5.43) | 7.65 | 5.13 (4.26-6.12) |
| 50-59 | 15.55 | 2.52 (2.33-2.71) | 4.67 | 2.29 (2.01-2.60) | 3.46 | 2.15 (1.85-2.49) | 6.57 | 2.97 (2.63-3.34) |
| 60-69 | 14.30 | 1.71 (1.61-1.80) | 6.23 | 1.74 (1.59-1.89) | 3.14 | 1.55 (1.39-1.73) | 5.10 | 1.94 (1.75-2.15) |
| 70-79 | 13.36 | 1.44 (1.37-1.51) | 6.56 | 1.45 (1.35-1.56) | 3.71 | 1.48 (1.34-1.64) | 3.30 | 1.48 (1.33-1.64) |
| >80 | 15.36 | 1.41 (1/32-1.51) | 5.60 | 1.30 (1.18-1.42) | 5.56 | 1.67 (1.47-1.90) | 5.22 | 1.71 (1.49-1.95) |
| **Sex** |  |  |  |  |  |  |  |  |
| Male | 15.54 | 1.70 (1.63-1.76) | 5.86 | 1.65 (1.55-1.75) | 3.86 | 1.62 (1.50-1.74) | 5.76 | 1.94 (1.81-2.08) |
| Female | 15.22 | 1.78 (1.71-1.85) | 6.30 | 1.64 (1.54-1.74) | 3.91 | 1.86 (1.71-2.02) | 5.0 | 2.25 (2.07-2.43) |
| **Race** |  |  |  |  |  |  |  |  |
| Black | 26.24 | 2.26 (2.08-2.45) | 9.10 | 1.93 (1.70-2.20) | 6.47 | 2.19 (1.85-2.56) | 4.56 | 1.88 (1.76-1.99) |
| White | 14.30 | 1.66 (1.61-1.72) | 5.90 | 1.59 (1.52-1.67) | 3.78 | 1.70 (1.60-1.81) | 10.71 | 3.39 (2.93-3.91) |
| American Indian/ | 27.63 | 3.11 (1.97-4.66) | 13.12 | 4.11 (1.97-7.55) | 2.64 | 1.62 (0.44-4.14) | 9.73 | 3.35 (1.44-6.59) |
| Asian or Pacific Islander | 15.10 | 1.91 (1.74-15.10) | 5.28 | 1.91 (1.63-2.23) | 2.87 | 1.54 (1.28-1.83) | 7.09 | 2.44 (2.10-2.83) |
| Unknown | -10.58 | 0.36 (0.12-0.83) | -6.25 | 0.15 (0.00-0.86) | -3.06 | 0.27 (0.01-1.49) | 1.32 | 1.31 (0.43-3.06) |
| **SEER staging** |  |  |  |  |  |  |  |  |
| Localized | 12.54 | 1.59 (1.52-1.65) | 5.29 | 1.55 (1.45-1.64) | 2.69 | 1.49 (1.37-1.62) | 2.10 | 1.95 (1.81-2.10) |
| Regional | 16.57 | 1.79 (1.72-1.88) | 6.98 | 1.74 (1.63-1.86) | 4.45 | 1.83 (1.68-1.99) | 2.14 | 1.97 (1.81-2.14) |
| Distant | 25.44 | 2.52 (2.25-2.81) | 5.29 | 1.74 (1.41-2.12) | 9.33 | 3.07 (2.52-3.70) | 3.83 | 3.17 (2.61-3.83) |
| Unknown | 36.10 | 2.66 (2.27-3.10) | 9.95 | 2.01 (1.57-2.53) | 2.98 | 2.23 (1.63-2.98) | 2.72 | 3.52 (2.72-4.48) |
| **Surgery** |  |  |  |  |  |  |  |  |
| No | 41.65 | 3.39 (2.94-3.88) | 13.42 | 2.69 (2.13-3.34) | 7.47 | 2.69 (1.96-3.60) | 20.48 | 5.71 (4.61-6.99) |
| Yes | 18.81 | 2.01 (1.94-2.08) | 6.56 | 1.77 (1.67-1.88) | 5.00 | 2.07 (1.93-2.22) | 6.91 | 2.51 (2.35-2.68) |
| Unknown | -14.88 | 0.00 (0.00-10.13) | -7.92 | 0.00 (0.00-10.96) | -4.35 | 0.00 (0.00-19.94) | -4.30 | 0.00 (0.00-20.16) |
| **Pathology grade** |  |  |  |  |  |  |  |  |
| Well differentiated | 15.53 | 1.72 (1.58-1.88) | 7.66 | 1.79 (1.57-2.03) | 4.42 | 1.80 (1.51-2.13) | 3.50 | 1.67 (1.39-1.99) |
| Moderately differentiated | 14.38 | 1.67 (1.61-1.73) | 5.53 | 1.57 (1.49-1.66) | 3.79 | 1.69 (1.57-1.81) | 5.06 | 1.97 (1.84-2.11) |
| Poorly differentiated | 19.12 | 1.91 (1.77-2.05) | 2.06 | 1.85 (1.65-2.06) | 5.33 | 2.00 (1.73-2.30) | 5.19 | 2.03 (1.75-2.34) |
| Undifferentiated | 11.80 | 1.61 (1.06-2.34) | 2.92 | 1.74 (0.95-2.92) | 2.13 | 1.46 (0.53-3.17) | 0.05 | 1.01 (0.28-2.59) |
| Unknown | 16.333 | 1.75 (2.06) | 1.87 | 1.65 (1.45-1.87) | 2.54 | 1.53 (1.28-1.81) | 8.88 | 2.92 (2.57-3.30) |
| **Calendar year** |  |  |  |  |  |  |  |  |
| 1992-2002 | 15.71 | 1.64 (1.56-1.72) | 7.41 | 1.70 (1.58-1.82) | 4.25 | 1.63 (1.48-1.78) | 4.28 | 1.70 (1.55-1.87) |
| 2003-2012 | 15.22 | 1.80 (1.73-1.86) | 5.43 | 1.61 (1.53-1.70) | 3.70 | 1.79 (1.66-1.92) | 5.94 | 2.29 (2.15-2.44) |
| **Follow-up time** |  |  |  |  |  |  |  |  |
| 0-4 | 26.10 | 2.28 (2.20-2.37) | 9.01 | 2.01 (1.90-2.13) | 7.49 | 2.38 (2.22-2.54) | 9.35 | 2.84 (2.66-3.02) |
| 4-8 | 7.74 | 1.36 (1.28-1.45) | 3.84 | 1.40 (1.28-1.52) | 1.04 | 1.19 (1.05-1.35) | 2.96 | 1.57 (1.40-1.76) |
| >8 | 1.67 | 1.08 (1.00-1.16) | 2.54 | 1.25 (1.13-1.37) | -0.36 | 0.93 (0.79-1.09) | 1.16 | 0.99 (0.84-1.16) |

Abbreviation: AER, absolute excess risk; SIR, standardized incidence ratio; SPCRC, secondary primary colorectal cancer; RCC, right-sided colon cancer; LCC, left-sided colon cancer.

**Figure S1**. Temporal trend of secondary primary colorectal cancer (SPCRC) incidence over the 22-year follow-up period (A). Trend of SPCRC incidence per 1000 person-years is estimated by Joinpoint regression. ^ P < 0.05. The solid black line shows the trend in (1) right-sided colon cancer, RCC; (2) left-sided colon-cancer, LCC (3) Rectosigmoid cancer in the United States from 1992 to 2012. The X-axis represents the intervals of follow-up and the Y-axis represents rates per 100,000 of the US population.
